# Non-Linear Effects of the Built Environment and Social Environment on Bus Use among Older Adults in China: An Application of the XGBoost Model

**DOI:** 10.3390/ijerph18189592

**Published:** 2021-09-12

**Authors:** Lanjing Wang, Chunli Zhao, Xiaofei Liu, Xumei Chen, Chaoyang Li, Tao Wang, Jiani Wu, Yi Zhang

**Affiliations:** 1 State Key Laboratory of Ocean Engineering, China Institute for Urban Governance, Shanghai Jiao Tong University, Shanghai 200240, China; lanjing.wang@sjtu.edu.cn (L.W.); cyljjf@sjtu.edu.cn (C.L.); wangtao127@sjtu.edu.cn (T.W.); JiaNiZi@sjtu.edu.cn (J.W.); 2 Transport & Roads, Department of Technology and Society, Faculty of Engineering, Lund University, 221 00 Lund, Sweden; Chunli.Zhao@tft.lth.se; 3 Key Laboratory of Advanced Public Transportation Science, China Academy of Transportation Sciences, MOT, Beijing 100029, China; liuxf@motcats.ac.cn (X.L.); chenxm@motcats.ac.cn (X.C.)

**Keywords:** non-linear, built environment, social environment, bus use, older adults, XGBoost model, threshold effect

## Abstract

Global aging has raised increasing concerns on the health and well-being of older adults. Public transport is a viable option to improve the mobility and quality of life among older adults. However, policies that promote the public transport use among older adults are rare. This study utilizes the eXtreme Gradient Boosting (XGBoost) decision tree to explore the non-linear associations of the built and social environment with bus use among older adults in China. The bus use of older adults was obtained from the Zhongshan Household Travel Survey (ZHTS) in 2012. Results show that non-linear relationships exist among all built environment and social environment characteristics. Within certain thresholds, the percentage of green space land use, land use mixture, bus-stop density, and dwelling unit density are positively related to bus use among older adults. Likewise, one social environment variable, the proportion of older adults in a neighborhood, is the key social environment variable. Furthermore, the dwelling unit density and proportion of older adults appear to have an inverse U-shaped relationship. Additionally, age, ownership of motorcycles, and distance from home to the nearest bus stop also show non-linearity. The findings presented in this paper facilitate effective planning interventions to promote bus use among older adults.

## 1. Introduction

Driven by the increase in life expectancy and the decline in fertility, the age structure of the world’s population continues to undergo sustained changes. The proportion and number of older adults in the total population are increasing dramatically [1]. The Population Division of the United Nations Department of Economic and Social Affairs (UN DESA) pointed out that in 2020, the global population of 65 and over is estimated to be 727 million, and this number is projected to more than double to over 1.5 billion by 2050 [2]. One of the goals of the 2030 Agenda for Sustainable Development is to ensure healthy lives and promote well-being for all at all ages [3]. This pledge implies that all segments of society and all ages must be considered, with special attention to the most vulnerable groups, including older adults. In this context, the living arrangements for older adults are increasingly important to policymakers. It is imperative for planners to grasp the distinctive travel patterns of older adults, especially in countries with advanced population aging [4]. Previous studies have highlighted that continuous mobility has a strong correlation with active aging and the health status, well-being, and quality of life of older adults [5,6,7]. Therefore, it is necessary to provide basic mobility for all people [8], especially for the “transportation disadvantaged” populations (e.g., physically disabled, older people, without a motor vehicle, and disadvantage caused by location) [9].

For older adults in China, active travel is essential to ensure participation in civil and social life [10]. Public transport is not only a viable alternative to meet the mobility needs of older adults, but it also contributes to promote transportation-related walking and facilitates physical activity of community-dwelling older adults [11,12,13]. Walking, as a form of active travel, is a beneficial source of physical activity to maintain health for older adults [14]. The experience during the travel, and a series of activities in the travel destination, can further improve life satisfaction [15]. In addition, recent studies have demonstrated that compared with young people, physical activity has a greater impact on the cognitive ability of older adults [16]. It can effectively prevent cognitive decline [17] and reduce the risk of obesity, diabetes, high blood pressure, and other diseases [18]. Hence, providing a better public transport environment and encouraging older adults to use public transport is a vital part of improving their mobility and quality of life [19]. In addition, gender differences are widespread in travel mode choice and actual use. Males and females usually have different travel reasons and concerns, such as safety, economy, and physical strength. As the urbanization and the feminization of the urban labor force continues, public transportation systems become more and more important [20]. Besides, individuals tend to use more public transport as they get older, for both males and females [21]. Therefore, it is important to further explore the different influences of different factors on the bus use among male and female passengers and promote the equal access of males and females to public transport.

The “Public Transport Priority Strategy” in China was proposed in 2005. However, there are few relevant policies to promote public transport use among older adults. Presumably, there are two main reasons. First, few studies have studied the public transport travel behavior of older adults. Secondly, existing studies have simplified the non-linear relationship between the built environment and public transport travel behavior. Most studies employed linear regression models, such as negative binomial regression models [22], linear regression model [23], Tobit regression model [24], and propensity score matching model [25]. Recently, many scholars have attempted to disentangle the non-linear associations between the built environment and travel behaviors. The most used methods are the gradient boosting decision trees model (GBDT) and random forest model [26,27,28,29,30]. Compared with traditional linear models, the non-linear methods offer higher prediction precision and perform better in interpreting complex relationships. These studies support that the pre-specified relationship between the independent variables and dependent variable in the linear regression is flawed [30], and renders the non-linear relationship unobservable [31].

To fill these two gaps, this study employs the eXtreme Gradient Boosting decision tree (XGBoost), an improved version of the gradient boosting decision tree (GBDT), to disentangle the underlying non-linearity between the built and social environment and bus use among older adults in China. The study is the first attempt to quantitatively look at the non-linear relationships between various factors and bus use among older adults. The remainder of this paper is structured as follows. Section 2 reviews the relevant literature on travel behavior among older adults and the related linear and non-linear studies. This is followed by Section 3, which provides an overview of the data and variables. Section 4 introduces the modeling approach. Section 5 presents the results, discusses the key determinants, and suggests implications for planning practice. Section 6 recapitulates the major findings, contributions, and limitations.

## 2. Literature Review

Aging is a worldwide phenomenon that creates significant challenges for all societies. Scholars have recognized the significance of studying the built and social environment in affecting the travel behavior among older adults. However, most of the research focuses on walking [30,32,33,34] and cycling [18,35,36], or considering both walking and cycling [37,38]. Little attention has been paid to public transport use among older adults. Due to the differences in cultural background, economic development level, and travel pattern, older adults in China have little car dependence compared with their counterparts in developed countries [39]. Thus, public transport, especially the bus, is one of the most feasible travel modes for older adults in China, especially for those who cannot afford a car or have stopped driving [5]. Compared with cycling and walking, public transport can help older adults to reach farther places, participate in more life and social activities, and avoid social isolation [40]. Existing literature on public transport use among older adults mainly explores the impact of bus fares [39], public transportation-related walking [41], and cycling [42]. The research on the frequency and duration of public transport trips of older adults is rare. Considering the importance of public transport in the lives of older adults, we hope that the research results can promote the changes beneficial to older adults, and provide better support for decision-making.

In view of the existing gaps, Broome, McKenna, Fleming, and Worrall [40] proposed six vital research directions for future research on bus usage for older adults in their review article. For example, they suggested to consider all the environments involved in the trip chain and perspectives of older adults, systematically analyze the facilitators and obstacles to public transport use among older adults, and quantitatively measure the relative importance of each environmental factor [40]. Most of the studies focus on the relationships between the built environment and travel behavior. The built environment characteristics are mainly derived from the “five Ds” proposed by Ewing and Cervero [43]. Some studies also indicated that greenery and aesthetically pleasing scenery have a positive effect on promoting the public transport use and active travel of older adults [44]. Other related research focused on personal, attitudinal, household, and social environment attributes [18,22,45].

In addition, in terms of research methods, previous studies tend to assume a pre-defined linear relationship between the independent and dependent variables. Yang et al. [46] utilized linear regression and logistic regression models to compare the data of the older adults and the middle-aged adults in the 2009 National Household Travel Survey (NHTS). They studied the active travel and public transport use among older adults, as well as the characteristics of the built environment associated with them. Based on data from Singapore in 2018, Song, Yap, Hou, and Yuen [18] adopted a simultaneous equation approach to disentangle the complex inter-relationships between built environment attributes, physical health, and physical activity of older adults. Zhang, He, Wu, and Li [22] used the negative binomial regression models, a continuous mixed Poisson distribution, to explore the impact of five categories of variables on public transport use among older adults. Other linear regression models employed in similar studies include probit regression [47], the multiple discrete continuous extreme value model, and logistic regression [48]. In particular, Table 1 summarizes the previous studies on the built environment and public transport and the methods applied. The results of the linear models simply reveal the positive or negative relationships between variables. Vergel-Tovar and Rodriguez [49] found that mixed land use, pedestrian infrastructure, and public facilities around the stations are positively associated with ridership, while the long distance to the CBD has a negative effect. Regarding the modal choice of public transit, Yu et al. [50] found that mixed land use has an adverse effect, while the increasing bus stops and residential density generated a positive effect. Few studies have employed nonlinear models to explore the association between the built environment and public transport use. Lin et al. [51] found that the ridership increases up to about 1000 trips when the entropy index of land use mixture exceeds the threshold of 0.6, below which it has a minuscule influence. Most variables display complex non-linear and threshold effects on ridership in their research. In the results of Ding et al. [52], all built environment variables display non-linear relationships with transit commuting. For example, distance to CBD is positively related to the likelihood of choosing transit, within the range of 0–8 km. When the intersection density exceeds the threshold of 12 intersections/km^2^, it is negatively associated with the likelihood.

Over the last decade, scholars have questioned these linear or predefined relationships. One of the most important and challenging research questions raised by van Wee and Handy [53] is: “To what extent are these effects non-linear?” Galster [54] once explored and summarized the non-linearity and threshold effects conceptually. The non-linear effect refers to when the predictors–response relationship within the domain of all variables is out of proportion, and the threshold effect is “a special case of non-linear effect”, in which the marginal value–response relationship will change suddenly at the threshold point [54]. Recently, studies began to assume the non-linear relationships between the built environment and travel behavior and attempted to discover the threshold effects. For example, using Shenzhen as the case study, Yang, Cao, and Zhou [28] utilized the gradient boosting decision tree (GBDT) to examine the irregularly non-linear associations between the built environment and urban vitality. The “urban vitality” there can be understood as the movement of people across urban spaces and is measured by the Baidu Heat Index [28].They found that the associations change remarkably beyond some thresholds of the built environment and that there exist non-linear synergies [28]. Tu et al. [55] used GBDT to study the non-linear effects and the thresholds of the built environment on ride-splitting based on partial dependence plots in the context of Chengdu. By combining with practical examples, Ding, Cao, and Naess [26] expounded the advantages of GBDT more vividly and concretely. Yin et al. [56] theoretically proposed four advantages of GBDT compared with traditional linear regression. This study introduces the XGBoost modeling approach, an improved version of GBDT, to test the hypothesis and disentangle the complex non-linear relationships between five categories of independent variables and public transport use among older adults in China.

It is worth mentioning that this paper utilized the same dataset and independent variables as Zhang, He, Wu, and Li [22]. However, the two studies differ remarkably in modeling approach and contribution. Zhang, He, Wu, and Li [22] chose a negative binomial regression model and assumed that all variables have exponential correlations with bus use among older adults. That research design is commonly seen in the literature. However, the present study employed the XGBoost approach and hypothesized that all variables have non-linear effects on the bus use among older adults. The results substantiated the hypotheses and challenged the conventional assumption of Zhang, He, Wu, and Li [22]. These findings will facilitate the interventions to promote public transport use among older adults and provide new evidence for the land use-travel literature. Furthermore, the results in the two papers are substantially different because of different distribution assumptions [57].

**Table 1:** Studies on the relationship between built environment and public transport.

Study	Sample (Area)	Dependent Variables	Built Environment	Method
Vergel-Tovar and Rodriguez [49]	120 BRT stations in seven cities (Colombia, Brazil, Guatemala, and Ecuador in Latin American)	BRT ridership	Density, Diversity, Design, Destination accessibility	Factor analysis, Cluster analysis, Log-linear regression
Li et al. [58]	124 subway stations (Guangzhou, China)	Rail transit ridership	Density, Diversity, Station characteristics	Geographically weighted regression (GWR), K-means clustering
Lin, Weng, Brands, Qian, and Yin [51]	1151 TAZs in the Sixth Ring Road of Beijing (Beijing, China)	Public transport ridership	Density, Design, Diversity, Distance	Light Gradient Boosted Machine (LightGBM)
Chakour and Eluru [59]	8000 stops in Montreal the ridership (Montreal, Canada)	Boardings/ Alightings per hour	Design, Distance to transit, Diversity, Destination accessibility	Composite Marginal Likelihood (CML)-based ordered response probit (ORP) model
Chen et al. [60]	Four weeks of smart card data (Nanjing, China)	Intermodal transit trips (bus and metro)	Density, Diversity, Design, Distance to transit, Destination accessibility	Traditional random forest incorporates the GWR model
De Gruyter et al. [61]	10,289 SA1s in Metropolitan Melbourne (Melbourne, Australia)	Commuting trips by transit/ train/ tram/bus (all modes)	Density, Diversity, Design, Destination accessibility, Distance to transit, Demand management	Ordinary least squares (OLS), Beta regression
Yang, Xu, Rodriguez, Michael, and Zhang [46]	75,862 older adults; 104,613 adults aged between 45 and 64 (U.S.)	Public transport trips	Design, Destination accessibility	Linear regression, Logistic regression
Zhao et al. [62]	Approximately 3,000,000 daily card-swiping records of transit users in March 2015 (Wuhan, China)	Transit trip rates	Density, Diversity, Distance to the bus stop, Distance to the destination	Bilevel hierarchical linear model (HLM)
Liu et al. [63]	Go card data containing trip transactions of all commuters using bus, train, and ferry services for two one-week periods (21–27 March 2016 under the old fare policy, and 20–26 March 2017 under the new fare policy) (South East Queensland, Australia)	The change in public transport ridership	Diversity, Density, Destination accessibility, Distance,	Spatial lag regression (SLR)
Ding, Cao, Yu, and Ju [52]	3758 commuters (Nanjing, China)	Transit commuting mode choice	Density, Diversity, Design, Destination accessibility, Distance to CBD	Semi-parametric multilevel mixed logit model
Yu, Xie, and Chan [50]	565 respondents from urban villages; 985 respondents from formal residences (Shenzhen, China)	Public transit choice	Density, Diversity, Distance to transit	Multinomial logistic regression (MNL)
Pongprasert and Kubota [64]	477 respondents (online questionnaire: 160; on-the-road survey: 317) (Bangkok, Thailand)	The probability with which car users’ switch to transit	Destination, Distance, Diversity, Density, Design, Demand management	Binary logistic regression model

## 3. Data

### 3.1. Study Area

**Figure 1:**
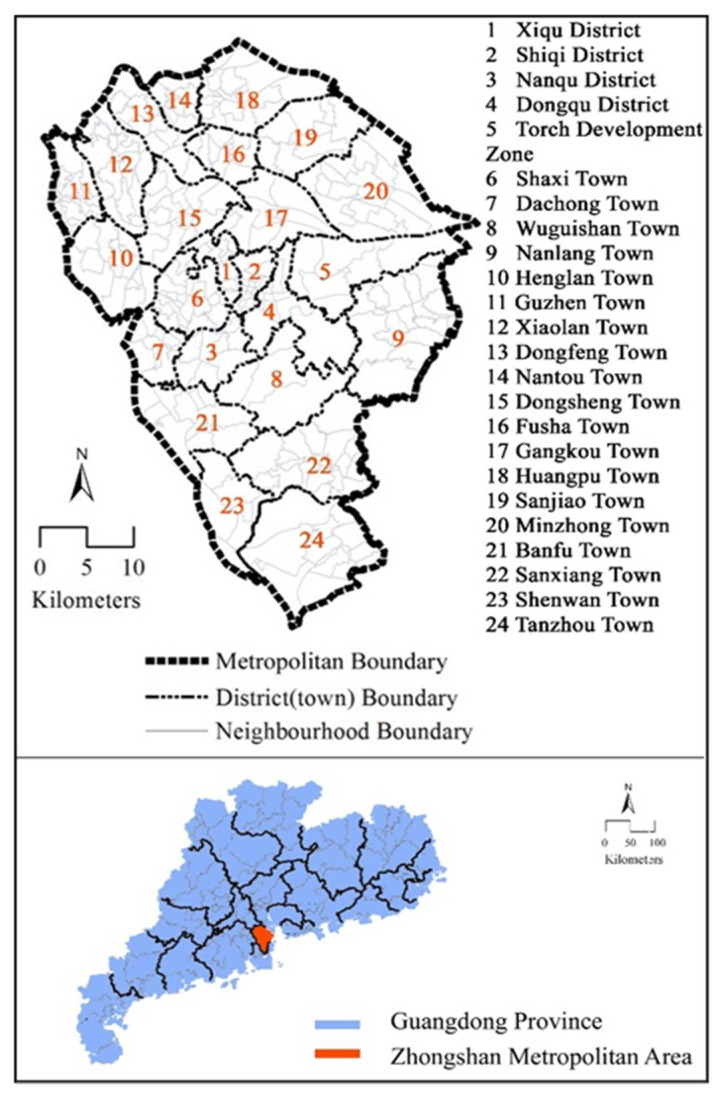
Study area.

This study chose Zhongshan in Guangdong province in China as a study case. Zhongshan is a medium-sized prefecture-level city with 24 towns [65]. It has a total land area of 1783.67 km^2^. and a population of about 4.42 million [66]. Zhongshan is an important city in the Guangdong-Hong Kong-Macao Greater Bay Area (GBA) and the Pearl River Delta (Figure 1). Among the three most competitive coastal city clusters in China, nearly 20 medium-sized cities have similar urbanization and motorization levels and transport characteristics to Zhongshan [19]. Hence, the findings in Zhongshan are expected to facilitate the interventions to bus use among older adults in similar cities.

By the end of 2018, Zhongshan had a total of 185 bus lines, 3740 bus stops, and 2288 buses. The modal split of buses among all travel modes and motorized travel modes are 4% and 6.8%, respectively [67,68]. The 300 and 500 m radius service coverage in the whole city are 46.81% and 77.7%, respectively. In the urban area, the 500 m radius service coverage is 88.93% [69].

### 3.2. Data Collection

The data used in this study were from the Zhongshan Household Travel Survey (ZHTS) in 2012, collected by the Zhongshan Urban Planning Bureau. This study used the same dataset as Zhang, He, Wu, and Li [22]. The ZHTS adopted the stratified random sampling method. It contained a self-reported travel diary, personal and household socio-demographics, and attitudes towards different travel modes [22]. We selected the respondents aged 60 years and above [30] and the sample size was 4329 individuals. It included 2616 males and 1713 females from 60 to 95 years old. Among the respondents, 491 (11.3%) took at least one bus trip per day.

### 3.3. Variables

This study adopted the frequency of daily bus trips as a proxy for the bus use among older adults. Trip frequency is one of the main indicators in attaining the recommended physical activity level. A higher level of bus use often means a greater likelihood of completing the required physical activity [70]. This study selected five categories of independent variables. They are personal, attitudinal, household, built environment, and social environment attributes. The built environment refers to the human-made environment where we live and work and measures the objective physical environment features, while the social environment consists of the individuals who we interact with and the culture that we live in [22]. The socio-demographics refers to the age, sex, household income, ownership of cars, motorcycles, bikes, and e-bikes, etc. In this study, the built and social environment variables are calculated at the aggregate neighborhood level, and the socio-demographic characteristics at the individual or household level.

#### 3.3.1. Characterization of Personal, Attitudinal, and Household Variables

The personal attributes include gender and age. The attitudinal attributes represent the preference for different travel modes, including walking, bicycle, bus, electric-bike, motorcycle, and car [46]. The household attributes include the household size, income level, ownership of different vehicle instruments, and the distance to the nearest bus stop.

#### 3.3.2. Characterization of Social Environment and Built Environment Variables

The social and built environment variables were defined based on neighborhoods by Zhang, He, Wu, and Li [22]. In Zhongshan, a neighborhood is homogeneous in terms of socio-demographics and living conditions [71]. According to the administrative division of Zhongshan, the entire 274 neighborhoods were selected in this study. These neighborhoods cover 1783.67 km^2^. The average size of a neighborhood is 6.51 km^2^.

Social environment is reflected by segments of the population with different socio-demographic characteristics, e.g., age structure, average income, and average education level [72]. Hypothetically, the perception and actual use of public transport among older adults may differ in different social environments [22]. In this study, we characterized two types of social environment attributes, i.e., the proportions of the older population and the proportions of high (medium or low)-income households. These attributes will indicate the social environment of the neighborhood where older adults live.

The built environment attributes in this study are derived from the “five Ds” proposed by Ewing and Cervero, namely density, diversity, design, distance to transit, and destination accessibility [43]. The “five Ds” have been widely used in built environment-travel studies. Zhang used them to study the relationship between the built environment and the frequency of cycling trips among older adults [73]. Ding chose four Ds to investigate the non-linear effects of built environment attributes on Metrorail ridership [27]. According to the definition of “five Ds”, we chose five corresponding variables (Table 2). In addition, greenery and aesthetically pleasing scenery have a positive effect on the physical activity among older adults [44]. Hence, we add the sixth built environment attribute, the percentage of green space land use (GREENSPACE).

The calculations of DWELLING, INTERSECTION, BUSSTOP, and GREENSPACE are explained in Table 3. The MIXTURE represents the degree to which different land uses are mixed in a neighborhood. We used the entropy index (*EI*) to measure land-use mixture [74]. *EI* is defined as follows.

(1) $$EI=\sum_{i=1}^{n}P_{i}\log\left(1/P_{i}\right)$$

In our context, *P_i_* represents the percentage of land use *i*’s coverage over total land use coverage. *n* represents total number of unique land use types with a value of not less than 1. When *EI* changes from 0 to 1, the land use level changes from a single-use environment to a multi-use balanced development environment [65].

**Figure 2:**
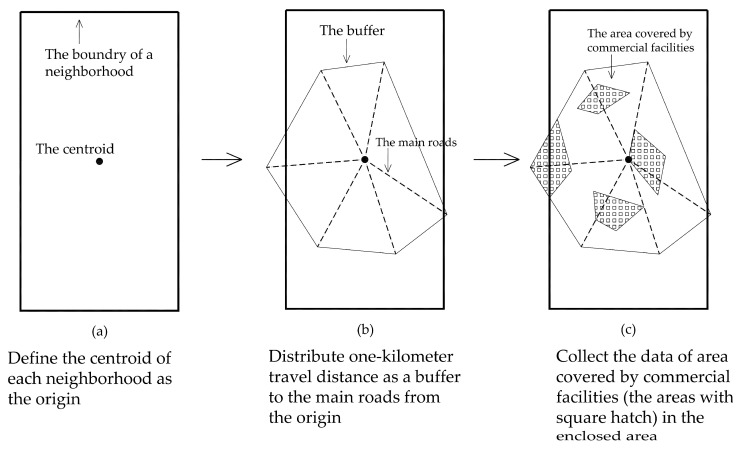
The calculation of commercial accessibility.

The 2012 Zhongshan Household Travel Survey (ZHTS) shows that a travel distance of one kilometer covers 70% of older adults’ home-based commercial trips. Therefore, we defined COMMERCIAL as the area of coverage of commercial facilities within a one-kilometer distance from the centroid of a neighborhood. It represents the ease of access to commercial attractions. For each neighborhood, the steps to achieve commercial accessibility are shown below (Figure 2):

1. Define the centroid of each neighborhood as the origin,
2. Distribute a one kilometer travel distance as a buffer to the main roads from the origin,
3. Form an enclosed area with the endpoints of the acceptable travel distances in ArcGIS,
4. Collect the data of the area covered by commercial facilities in the enclosed area in ArcGIS,
5. Divide the data by the population of the neighborhood to obtain the commercial accessibility.

## 4. Method

This study introduced an improved GBDT method, namely XGBoost, to analyze the possible non-linearity. GBDT was originally developed by computer scientists to predict and interpret data [75]. In recent years, GBDT has been innovatively adopted to explain the non-linear effects of built environmental attributes on travel [26]. XGBoost and GBDT are both a kind of boosting machine learning method. XGBoost is derived from GBDT, but it strives to maximize speed and efficiency. Therefore, it is called X (extreme) GBoost [76]. The XGBoost algorithm usually performs better compared with other algorithms applied [77]. Wang argued that XGBoost and its variations will remain one of the most widely used methods in the data science community in the near future [78]. The approach has several advantages compared with the traditional model and the GBDT model [79]:

- Regularization term: XGBoost adds the regularization term to control the complexity of the model. It helps to prevent overfitting and improve the generalization ability of the model.
- Second-order derivative: GBDT only uses the first-order derivative information of the cost function in the model training. XGBoost performs a second-order Taylor expansion on the cost function, and both the first and second derivatives can be used.
- Column sampling: The traditional GBDT uses all the data in each iteration. XGBoost uses a strategy similar to the random forest. It supports data sampling and column sampling, which not only reduces overfitting, but also reduces calculations.
- Missing value processing: The traditional GBDT is not designed to deal with missing values. XGBoost can automatically learn its splitting direction.

The main sources of error of XGBoost are: (1) training error and (2) model complexity [76].

The loss function can be defined as:

(2) $$obj\;=\;\sum_{i=1}^{n}l\left(y_{i},\hat{y}\right)\;+\;\sum_{i=1}^{k}\Omega \left(f_{k}\right)$$

Then the objective function can be written as:

(3) $$obj^{\left(t\right)}\;=\;\sum_{i=1}^{n}l\left(y_{i},{\hat{y}}^{\left(t-1\right)}\;+\;f_{t}\left(x_{i}\right)\right)\;+\;\Omega \left(f_{t}\right)\;+\;constant$$

The objective function can be rewritten according to Taylor expansion:

(4) $$f\left(x\;+\;\Delta t\right)\;⋍\;f\left(x\right)\;+\;f^{\prime }\left(x\right)\Delta x\;+\;\frac{1}{2}f^{\prime \prime }\left(x\right)\Delta x^{2}$$

(5) $$obj^{\left(t\right)}\;⋍\;\sum_{i=1}^{n}\left[l\left(y_{i},{\hat{y}}^{\left(t-1\right)}\right)\;+\;g_{i}f_{t}\left(x_{i}\right)\;+\;\frac{1}{2}h_{i}f_{t}^{2}\left(x_{i}\right)\right]\;+\;\Omega \left(f_{t}\right)\;+\;constant$$

In further calculations, we do not need to consider the constant term, that is, the difference between the true value and the predicted value in the previous round.

(1) For the training error part, where $g_{i}$ represents the first derivative and $h_{i}$ represents the second derivative, the objective function only depends on the first derivative and the second derivative of each data point in the error function.

(6) $$g_{i}\;=\;\partial_{{\hat{y}}^{\left(t-1\right)}}l\left(y_{i},{\hat{y}}^{\left(t-1\right)}\right)$$

(7) $$h_{i}\;=\;\partial_{{\hat{y}}^{\left(t-1\right)}}^{2}l\left(y_{i},{\hat{y}}^{\left(t-1\right)}\right)$$

(2) When considering the complexity of the model, the complexity of a tree can be defined as follows:

(8) $$f_{t}\left(x\right)\;=\;\omega_{q\left(x\right)},\omega \in R^{T},q:R^{d}\to \left\{1,2,\ldots ,T\right\}$$

(9) $$h_{i}\;=\;\partial_{{\hat{y}}^{\left(t-1\right)}}^{2}l\left(y_{i},{\hat{y}}^{\left(t-1\right)}\right)\Omega \left(f_{t}\right)=\gamma T+\frac{1}{2}\lambda \sum_{j=1}^{T}\omega_{j}^{2}$$

where γ represents the number of leaves, *T* is the number of leaf nodes, and *w* represents the weight of the leaves. Then, the error function can be rewritten again as:

(10) $$\begin{array}{cc} obj^{\left(t\right)} & ⋍\;\sum_{i=1}^{n}\left[g_{i}f_{t}\left(x_{i}\right)+\frac{1}{2}h_{i}f_{t}^{2}\left(x_{i}\right)\right]+\Omega \left(f_{t}\right)\\ & =\sum_{i=1}^{n}\left[g_{i}\omega_{q\left(x_{i}\right)}+\frac{1}{2}h_{i}\omega_{q\left(x_{i}\right)}^{2}\right]+\gamma T+\frac{1}{2}\lambda \sum_{j=1}^{T}\omega_{j}^{2}\\ & =\sum_{j=1}^{T}\left[\left(\sum_{i\in I_{j}}g_{i}\right)\omega_{j}+\frac{1}{2}\left(\sum_{i\in I_{j}}h_{i}+\lambda \right)\omega_{j}^{2}\right]+\gamma T \end{array}$$

Define $G_{i}$ as the sum of the first degree of each leaf node, and $H_{i}$ as the sum of the second degree of each leaf node.

(11) $$obj^{\left(t\right)}=\sum_{j=1}^{T}\left[G_{j}\omega_{j}+\frac{1}{2}(H_{j}+\lambda )\omega_{j}^{2}\right]+\gamma T$$

Find the partial derivative, then:

(12) $$obj^{\left(t\right)}=-\frac{1}{2}\sum_{j=1}^{T}\frac{G_{j}^{2}}{H_{j}+\lambda }+\gamma T$$

**Figure 3:**
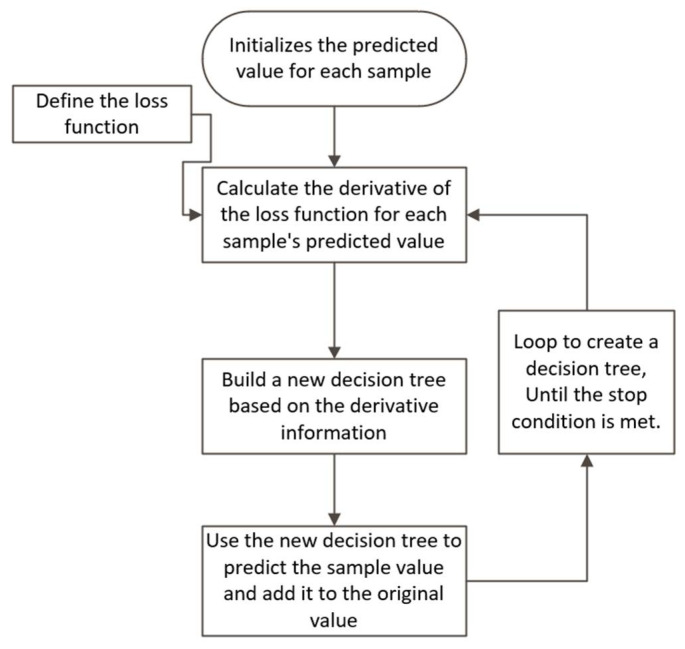
XGBoost algorithm flow chart.

Its algorithm can be summarized as follows (Figure 3):

First, add a new tree in each iteration.

Then, at the beginning of each iteration, calculate the first derivative $g_{i}$ and the second derivative $h_{i}$.

Next, use the statistics to greedily grow a tree $f_{t}\left(x\right)$:

(13) $$obj^{\left(t\right)}=-\frac{1}{2}\sum_{j=1}^{T}\frac{G_{j}^{2}}{H_{j}+\lambda }+\gamma T$$

where obj is the optimal total loss when the structure of the tree is determined.

Add $f_{t}\left(x\right)$ to the model ${\hat{y}}_{i}^{\left(t\right)}={\hat{y}}_{i}^{\left(t-1\right)}+f_{t}\left(x_{i}\right)$, and in order to avoid overfitting, a reduction factor “ϵ” is added:

(14) $$y^{\left(t\right)}=y^{\left(t-1\right)}+\epsilon f_{t}\left(x_{i}\right)$$

where ϵ is called step-size or shrinkage, usually set around 0.1, which means we do not perform full optimization in each step and reserve chance for future rounds, and it can help prevent overfitting.

## 5. Results and Discussion

This study used the “xgboost” package in Python to help develop the entire model [55,78,80]. We provided the relative importance ranking of all predicting variables to identify the significant correlates. Then, based on partial dependence plots, we illustrated the non-liner relationships and threshold effects with the different categories of variables.

### 5.1. Relative Importance of Independent Variables

Table 4 shows the relative importance of all the independent variables in the form of percentage, and their contributions amount to 100%. The relative importance of these independent variables represents their empirical improvements in reducing predicted error [56]. The ranking of these independent variables is derived according to the size of their relative importance. Among the five categories of independent variables, the built environment attributes contributed the most in predicting the bus trips of older adults. This echoes the results of similar studies using non-linear modeling methods [30]. Specifically, built environment characteristics collectively contribute to 41.97% of the prediction, higher than the personal socio-demographics (15.56%), attitudinal factors (6.59%), household socio-demographics (26.46%), and social environment characteristics (10.38%).

**Figure 4:**
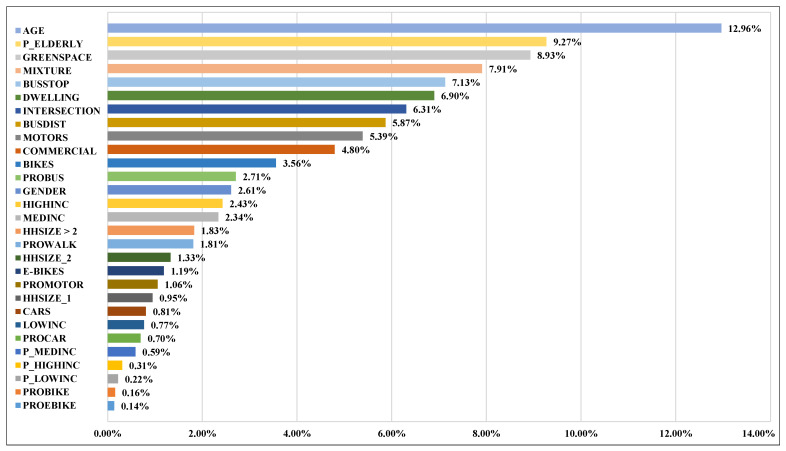
The ranking of the relative importance of independent variables.

All 6 built environment factors are ranked among the top 10 with respect to the relative importance (Figure 4). The percentage of green space land use among all land uses (8.93%, ranked 3rd) shows the largest contribution among the six built environment variables. The land-use mixture, bus-stop density, dwelling unit density, intersection density, and commercial accessibility are ranked 4th, 5th, 6th, 7th, and 10th, respectively.

For personal attributes, the age of the respondents contributes the most (12.96%) among all variables, while the gender is ranked the 13th. Unlike other travel modes, especially car and bike, the gap of public transport use between males and females may not be as significant as expected [21], as the results indicated in the current study. In Zhongshan, the differences between the bus use among older males and females is subtle. The average frequency of daily bus trips of older males and females are 0.24 and 0.23, respectively. The average travel duration is 3.24 and 3.26 min per day, respectively.

For attitudinal attributes, only the preference for bus and walking are ranked among the top 20 (12th and 17th, respectively). Older adults living in a bus-friendly neighborhood will have access to more convenient bus services, which may help to increase their preference for bus. The increasing preference for bus would also encourage older adults to choose bus more frequently and improve their hope to live in a bus-friendly neighborhood [81]. This will further promote the formation of bus-friendly neighborhoods. The rankings of relative importance of other attitudinal variables are all beyond the top 20. For household attributes, the distance from home to the nearest bus stop, the ownership of motorcycles, and the ownership of bikes are ranked 8th, 9th, and 11th. For social environment, the proportion of older adults in a neighborhood has the greatest impact (9.27%), ranked 2nd among all variables. The proportion of households with high-, medium-, or low-income levels in the neighborhood has meticulous effects (all less than 1%).

### 5.2. Non-Linear Relationships with Built Environment Variables

**Figure 5:**
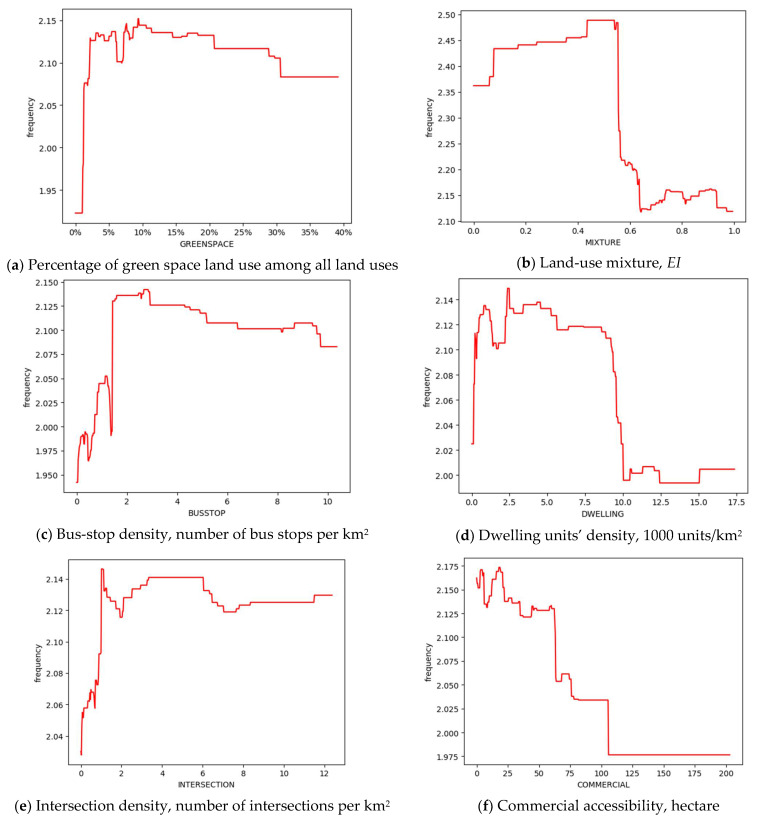
Associations between built environment variables and bus trip frequency among older adults in Zhongshan.

The partial dependence plot in Figure 5 depicts the effects of the built environment on bus use among older adults. They are illustrated in the order of their relative importance. We will focus on the four most important built environment factors, which are the percentage of green space land use, land use mixture, bus-stop density, and dwelling unit density.

#### 5.2.1. Non-Linear Relationship with the Percentage of Green Space Land Use

In Figure 5a, when the percentage of green space land use among all land uses is within the range of 2% and 9%, it is significantly related to the bus use among older adults. When it exceeds 20%, the positive effect is trivial. Beyond the threshold of 30%, the bus use even declines. China’s national standard of green space design points out that urban green space is beneficial to improve urban ecology, protect the environment, and provide residents with recreational destinations [82]. Previous studies showed that green space has a significant and positive influence on the active travel and bus use among older adults [44]. Our results further indicate that proper proportion (within 30%) of green space among all land uses does have a promoting effect.

#### 5.2.2. Non-Linear Relationship with the Land Use Mixture

In Figure 5b, when the *EI* of land use mixture changes from 0 to 0.43, the bus use rises steadily to the highest level, 2.49 trips per day. Then, the bus trip remains stable when the mixture is between 0.43 and 0.55. Mixed land use development features abundant commercial, service, and even employment opportunities and facilities. It increases the accessibility to various destinations, provides more options for participating in activities, and meets basic needs [81]. Therefore, mixed land use encourages bus use among older adults [10]. However, the land use mixture showed a negative association with bus use after the threshold of 0.55. When the land use becomes more mixed, a full range of clinics, parks, supermarkets, restaurants, and activity centers, etc., are near residents [80]. The majority of living, social, and entertainment needs of older adults can be satisfied in the vicinity of their homes. Different facilities have become physically or spatially adjacent because of the high level of land use mixture [10], which makes walking or cycling become attractive and feasible travel options, especially for older adults [10,57,83].

#### 5.2.3. Non-Linear Relationship with the Percentage of Bus-Stop Density

The result in Figure 5c suggests that 2.7 bus stops/km^2^ is the cutting point for the effect of bus-stop density. When the bus-stop density is lower than that threshold, the bus use among older adults will increase with extra bus stops. The possible reason is that denser bus stops makes it more convenient to access bus services. However, when the bus-stop density exceeds 2.7 bus stops/km^2^, the effect is saturated. It suggests that in neighborhoods with already high-level bus service provision, additional bus stops may not increase bus use among older adults.

#### 5.2.4. Non-Linear Relationship with the Dwelling Unit Density

The result in Figure 5d shows that the association between the dwelling unit density and bus use among older adults appears to be an inverse U-shaped relationship. The bus trip frequency increases sharply as the dwelling unit density rises from 20 to 500 units/km^2^. When the dwelling unit density continues to rise from 500 to 2500 units/km^2^, the bus use witnesses a subtle decrease. Following this, it becomes flat with slight fluctuations when dwelling unit density grows from 2500 to 8500 units/km^2^. That echoes previous studies that stated that high-density development facilitates bus use [10]. Beyond the threshold of 8500 units/km^2^, the dwelling unit density becomes ultra-high and the bus trip frequency declines dramatically. Ultra-high-density development is associated with more people taking buses, fewer seats, and more crowded carriages and bus stops. It will increases the risk of collision and injuries, especially for older adults [30]. The fear of falling and the poor bus service may limit the willingness of older adults to choose bus over other modes [5]. Therefore, in neighborhoods with ultra-high dwelling unit density, it is important to improve bus services and safety for older adults.

### 5.3. Non-Linear Relationships with Key Social Environment Variable

**Figure 6:**
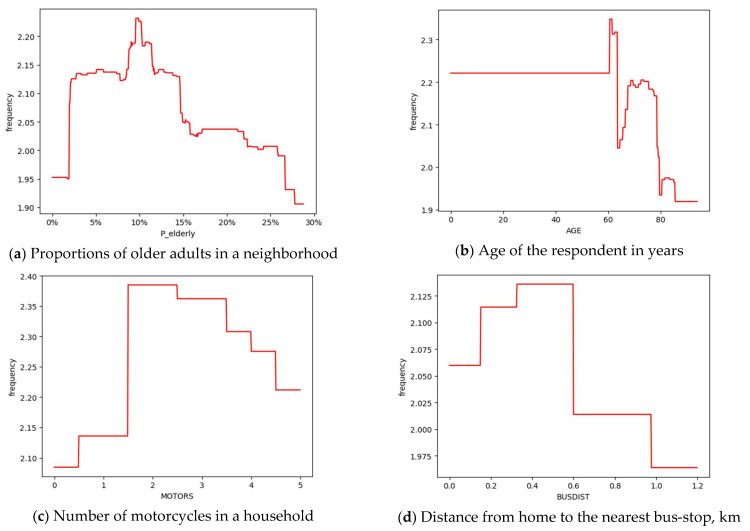
Associations between other key variables and bus trip frequency among older adults in Zhongshan.

The key social environment variable discussed is the proportion of older adults in a neighborhood. The relative importance of the variable is 9.27%, ranking 2nd among all variables. In Figure 6a, the frequency of bus trips peaks when the proportion of older adults in a neighborhood is within the range of 8% to 11%, and increases significantly. Compared with young adults, the population distribution of older adults is similar to the distribution of trip destinations [4]. Neighborhoods with higher proportions of older adults are more attractive to older adults [4]. However, beyond the threshold of 15%, the proportion of older adults demonstrates a certain inhibitory effect on bus use among older adults. The possible reason is that when the proportion is relatively high, older adults will spontaneously form groups to carry out a series of group activities. For Chinese older adults, the most prevailing group activities include square dancing, Tai Chi, playing mahjong, cards, chess, and so on [10]. It is not necessary to take a bus to a farther place for those activities.

### 5.4. Non-Linear Relationships with Key Personal and Household Variables

The key personal and household variables discussed are age, motorcycle ownership, and the distance from home to the nearest bus stop. The relative importance rankings of the three variables are 1st, 8th, and 9th, respectively.

#### 5.4.1. Non-Linear Relationships with Key Personal Variables

The result in Figure 6b shows that the bus use among older adults is gradually increasing between the ages of 60 and 67 and fluctuates slightly between the ages of 67 and 75. Older adults aged 75–85 take fewer bus trips and those over 85 years old use the bus even less. Older adults are not a homogenous group, differences exist between the ‘‘young’’ older adults and the ‘‘old’’ older adults in travel patterns and needs [84]. Mobility reductions become more obvious when people reach 80 years old [84]. Older adults are in a transition period as they will retire, no longer drive, and have lower income [4,11]. Therefore, they tend to choose safer, more convenient, and cheaper modes, e.g., bus, for medium- and long-distance travel [85].

#### 5.4.2. Non-Linear Relationships with Key Household Variables

The results in Figure 6c show an inverse V-shaped relationship between the household motorcycle ownership and bus use among older adults. The cutting point is 1.5 motorcycles per household. Within the cutting point, the bus use increases with the motorcycle ownership. Motorcycle is the most owned vehicle instrument in Zhongshan. It plays an important part in connecting older adults from home to bus stops. Older adults’ reliance on buses is partly related to the availability of family members to cater for their transport needs [84].

The results in Figure 6d also demonstrate an inverse V-shaped relationship between the distance from home to the nearest bus stop and bus use among older adults. Within the threshold of 600 m, the propensity of older adults to choose the bus is positively related to the distance from home to the nearest bus stop. However, the bus use among older adults declines when the distance exceeds 600 m. The physical strength required by the long distance to the nearest bus stop is a barrier for older adults. Other barriers include the safety concern when crossing roads with heavy traffic, poor pavement conditions, and lack of pedestrian crossings [5]. The national standard of China recommends that the distance between stops ranges from 500 to 800 m [86,87]. However, the results in Zhongshan indicate an effective range between 400 and 600 m for older adults. Therefore, in neighborhoods with high proportions of older adults, the bus stop distance is suggested to be within 400–600 m.

### 5.5. Model Comparison

#### 5.5.1. The Improvement of the XGBoost Model

It is worth noting that this study used the same dataset from Zhongshan City as Zhang, He, Wu, and Li [22]. However, the two studies are substantially different in research methods and results. Zhang, He, Wu, and Li [22] adopted the negative binomial regression model and assumed the linear relationships between the dependent variable and independent variables. However, our study used the XGBoost model, hypothesizing that built environment variables have non-linear associations with the frequency of bus trips among older adults. In the previous study, the land use mixture (MIXTURE), bus-stop density (BUSSTOP), and aesthetics (GREENSPACE) were the three most important variables in built environment attributes. This echoes our results. However, Zhang, He, Wu, and Li [22] claimed that more bus stops and more green space are positively correlated with bus use among older adults, while mixed land use is negatively correlated. For social environment attributes, their results indicated that older adults would use the bus more frequently if they live in a neighborhood with a smaller proportion of older population. However, the results are not able to determine how small the proportion should be. The assumption of linearity in variables in Zhang, He, Wu, and Li ’s study cannot capture the non-linear relationships between variables [31]. Compared with the previous study, the current study not only indicates whether the independent variable has a promotion or an inhibitory effect, but also depicts the changing curves and threshold effects of bus use frequency when the independent variable changes. It also confirms the hypothesis that nonlinear correlations may be universal and vary according to variables [57].

#### 5.5.2. The Performance of the XGBoost Model

As shown in Table 5, the value of testing pseudo-R² of the XGBoost model is 0.838. The corresponding value of the traditional multi-linear regression model is 0.160. Results show that the XGBoost model performs better than the traditional multi-linear regression model.

## 6. Conclusions

This study introduced the XGBoost model to explore the non-linear associations of the built and social environment with bus use among older adults in Zhongshan, China. Compared with studies using linear models, the results in the present study indicate the non-linearity and threshold effects of various factors. The results also confirmed the hypothesis that non-linear correlations are universal and vary according to the variables [57]. Policymakers and planners may facilitate policy-making processes and land use and transportation interventions to promote bus use among older adults.

Among the five categories of independent variables, the built environment attributes contributed the most in predicting the bus trips of older adults. All built environment variables play a substantial role in prediction. This further illustrates the efficacy of increasing bus use among older adults through land use and transportation planning. In Zhongshan, for example, appropriate amount of green space, convenient bus service, and moderately mixed development are connected with high frequency of bus trips among older adults. For Zhongshan, the effective thresholds of built environment intervention include: (1) the percentage of green space land use among all land uses, ranging from 2% to 9%, (2) the land use mixture entropy index between 0.43 and 0.55, (3) the cutting point of 2.7 bus stops/km^2^ for bus-stop density, and (4) the dwelling unit density ranging from 0 to 2500 units/km^2^.

For social environment variables, when the proportion of older adults in a neighborhood is beyond the threshold of 15%, it indicates a negative connection with bus use among older adults. Some personal and household variables also showed non-linearity. Older adults aged 60 to 75, 75 to 85, and over 85 years old demonstrate different bus use patterns. The suggested effective range of the distance from home to the nearest bus stop is within 400–600 m. Owning one or two motorcycles in a household is related to more bus use among older adults as motorcycles may connect older adults from home to bus stops. Although our results and recommendations may not be directly applicable to other cities or regions, the modeling approach developed in this study could be used in similar studies.

Favorable public policy can promote the development of public transport [88]. The above-mentioned findings could facilitate the understandings on how the built and social environment variables affect bus use among older adults [55]. It is informative for policymakers and planners to adopt reasonable and targeted interventions to improve bus use among older adults. First, the dwelling unit density of 8500 persons/km^2^ is sufficient to optimize bus use frequency among older adults in Zhongshan. When the proportion of older adults in a neighborhood exceeds the threshold of 15%, the over-aggregation of the older population may even have an inhibitory effect. Planners may consider developing neighborhoods with proper dwelling unit density and relatively balanced age structures [31]. Second, it is important to ensure an adequate green space land use coverage rate, but within an optimal range (preferably around 10%). When the rate is too high or too low, it will show negative correlations. Third, the results suggest promoting mixed land use development but avoiding excessive concentration of amenities. In addition, the location of bus stops should be optimized to better cater for older adults. Finally, planners should improve the facilities, services, and safety of the bus system to build bus-friendly neighborhoods and enhance the preference of older adults for bus use [89,90]. This study has a few limitations. First, XGBoost cannot generate confidence intervals for predictions and further research is needed to determine to what extent the importance of the variables is valid. Second, this study only focuses on one city in southern China (i.e., Zhongshan). Whether the findings can be generalized to other cities needs further study. Third, this study is based on cross-sectional data. At present, it is unable to confirm the potential causal effects of various factors.

## Figures and Tables

**Table 2:** The description of built environment variables.

Dimensions	Meaning	The Built Environment Variables Used in This Study
Density	The dwelling units or building floor area per unit of area.	Dwelling unit density (DWELLING)
Design	The street network characteristics within an area.	Intersection density (INTERSECTION)
Diversity	The number of different land uses in a fixed area and the represent degree	Land-use mixture (MIXTURE)
Distance to transit	The average of the shortest street routes from the residences or workplaces in an area to the nearest rail station or bus stop	Bus-stop density (BUSSTOP)
Destination accessibility	The ease of access to trip attractions	Commercial density (COMMERCIAL)
Aesthetic	The attractiveness and appeal of a place	Percentage of green space land use among all land uses (GREENSPACE)

**Table 3:** Definition and descriptive statistics of variables.

Variable	Definition	Mean/Percentage (%)	S.D.	Min.	Max.
Frequency	Frequency of bus trips among older adults, trips per day, count	0.27	0.73	0	6
Personal Variables					
GENDER	1 = Male	60.43	/	/	/
	0 = Female	39.57	/	/	/
AGE	Age of the respondent in years, count	67.05	6.61	60	95
Attitudinal Variables					
PROWALK	The respondent favors walking over other modes, binary, 1 = yes	26.80	/	0	1
PROBIKE	The respondent favors bicycle over other modes, binary, 1 = yes	16.49	/	0	1
PROEBIKE	The respondent favors e-bike over other modes, binary, 1 = yes	6.24	/	0	1
PROBUS	The respondent favors bus over other modes, binary, 1 = yes	22.89	/	0	1
PROMOTOR	The respondent favors motorcycle over other modes, binary, 1 = yes	12.04	/	0	1
PROCAR	The respondent favors car over other modes, binary, 1 = yes	2.75	/	0	1
Household Variables					
HHSIZE_1	Household size is one person, binary, 1 = yes	19.89	/	0	1
HHSIZE_2	Household size is two persons, binary, 1 = yes	35.34	/	0	1
HHSIZE > 2	Household size is three or more persons, binary, 1 = yes	44.77	/	0	1
HIGHINC	High household income (>60,000 RMB/year), binary, 1 = yes	15.22	/	0	1
MEDINC	Medium household income (20,000–60,000 RMB/year), binary, 1 = yes	47.82	/	0	1
LOWINC	Low household income (<20,000 RMB/year), binary, 1 = yes	36.96	/	0	1
BUSDIST	Distance from home to the nearest bus-stop (km), continuous	0.5	0.36	0.1	1.2
BIKES	Number of bikes in a household, count	0.61	0.71	0	5
E-BIKES	Number of electric bikes in a household, count	0.22	0.46	0	4
MOTORS	Number of motorcycles in a household, count	0.76	0.85	0	5
CARS	Number of private cars in a household, count	0.17	0.44	0	4
Social Environment Variables					
P_ELDERLY	Proportions of older adults in a neighborhood, continuous	0.14	0.06	0.01	0.29
P_HIGHINC	Proportions of high-income households in a neighborhood, continuous	15.64	/	0	1
P_MEDINC	Proportions of medium-income households in a neighborhood, continuous	61.21	/	0	1
P_LOWINC	Proportions of low-income households in a neighborhood, continuous	23.15	/	0	1
Built Environment Variables					
DWELLING	Dwelling units’ density, 1000 units/km^2^, continuous	3.34	4.32	0.02	17.42
INTERSECTION	Intersection density, number of intersections per km^2^, continuous	2.79	3.18	0	13.26
MIXTURE	Land-use mixture, Entropy Index, continuous	0.7	0.98	0	1
COMMERCIAL	Area coverage of commercial establishments within 1 km from the center of a neighborhood, in ha, continuous	33.19	33.08	0	230.46
BUSSTOP	Bus-stop density, number of bus stops per km^2^, continuous	0.7	0.18	0	1
GREENSPACE	Percentage of green space land use among all land uses, continuous	0.07	0.08	0	0.65

Note: Sample size = 4329; S.D. = Standard Deviation; Min. = minimum; Max. = maximum.

**Table 4:** Relative importance of independent variables in prediction.

Independent Variables	F Score	Relative Importance (%)	Ranking
Personal Variables		15.56%	
f0(GENDER)	136	2.61%	13
f1(AGE)	676	12.96%	1
Attitudinal Variables		6.59%	
f2(PROWALK)	101	1.81%	17
f3(PROBIKE)	9	0.16%	28
f4(PROEBIKE)	8	0.14%	29
f5(PROBUS)	151	2.71%	12
f6(PROMOTOR)	59	1.06%	20
f7(PROCAR)	39	0.70%	24
Household Variables		26.46%	
f8(HHSIZE_1)	53	0.95%	21
f9(HHSIZE_2)	74	1.33%	18
f10(HHSIZE > 2)	102	1.83%	16
f11(HIGHINC)	135	2.43%	14
f12(MEDINC)	130	2.34%	15
f13(LOWINC)	43	0.77%	23
f14(BUSDIST)	327	5.87%	8
f15(BIKES)	198	3.56%	11
f16(E-BIKES)	66	1.19%	19
f17(MOTORS)	300	5.39%	9
f18(CARS)	45	0.81%	22
Social Environment Variables		10.38%	
f19(P_ELDERLY)	516	9.27%	2
f20(P_HIGHINC)	17	0.31%	26
f21(P_MEDINC)	33	0.59%	25
f22(P_LOWINC)	12	0.22%	27
Built Environment Variables		41.97%	
f23(DWELLING)	384	6.90%	6
f24(INTERSECTION)	351	6.31%	7
f25(MIXTURE)	440	7.91%	4
f26(COMMERCIAL)	267	4.80%	10
f27(BUSSTOP)	397	7.13%	5
f28(GREENSPACE)	497	8.93%	3

**Table 5:** Comparison of the pseudo-R² between the XGBoost model and multi-linear regression.

	Model	XGBoost	Multi-Linear Regression
Metrix			
R²		0.838	0.160

## Data Availability

The dataset presented in this article is not readily available because it belongs to the Zhongshan Municipality Natural Resources and Planning Bureau and is a part of the ongoing projects (Grant No. 18BSH143 of the National Social Science Foundation of China, Grant No. 20692109900 and Grant No. 21692106700 of Shanghai Science and Technology Program, and Grant No. 2020-APTS-04 of APTSLAB). Therefore, the dataset is confidential during this period.
